# Dosimetric and hematological toxicity analyses of bone marrow‐sparing intensity‐modulated radiation therapy for patients with cervical cancer treated with extended‐field radiation therapy

**DOI:** 10.1002/pro6.70019

**Published:** 2025-06-12

**Authors:** Jia‐nan Wang, Xi Yu, Li‐na Gu, Dong‐mei Liu, Qiu‐yue Su, Jing‐qi Xia, Wei‐Kang Yun, Xin Li, Xue‐Yuan Hu, Shan‐Shan Yang, De‐Yang Yu

**Affiliations:** ^1^ Department of Gynecological Radiotherapy Harbin Medical University Cancer Hospital Harbin Heilongjiang China; ^2^ Department of Radiation Physics Harbin Medical University Cancer Hospital Harbin Heilongjiang China

**Keywords:** cervical cancer, bone marrow sparing, extended‐field  radiation therapy, hematological toxicity, intensity‐modulated radiation therapy

## Abstract

**Objective:**

This study aimed to assess the dosimetric parameters and hematological toxicity (HT) associated with bone marrow‐sparing (BMS) intensity‐modulated radiation therapy (IMRT) in patients diagnosed with International Federation of Gynecology and Obstetrics (FIGO) stage IIIC cervical cancer undergoing extended‐field radiation therapy (EFRT).

**Methods:**

Patients with cervical cancer presenting with common iliac or para‐aortic lymph node metastases require EFRT, which often results in grade 3 HT. Therefore, we retrospectively analyzed data of 84 patients with FIGO stage IIIC cervical cancer who underwent concurrent chemoradiotherapy (EFRT, brachytherapy, and weekly cisplatin 40 mg/m^2^) at Harbin Medical University Cancer Hospital, including 40 who received BMS‐IMRT and 44 who received normal IMRT. Dose–volume histogram (DVH) parameters and estimated treatment times were compared. We also compared acute HT between the normal and BMS groups.

**Results:**

Dosimetric analysis demonstrated that BMS‐IMRT significantly reduced the mean volume of bone marrow receiving ≥10, ≥20, ≥30, and ≥40 Gy without affecting the target coverage of planning target volume and sparing the organs at risk. Within the BMS‐IMRT group, 37.5% of the patients developed grade ≥3 HT, with an increase in HT (HT3+ = 61.4%) in patients receiving normal‐IMRT (*P* = 0.029).

**Conclusions:**

For patients with cervical cancer treated with EFRT, BMS‐IMRT represents a feasible treatment approach that may mitigate HT and facilitate the uninterrupted administration of concurrent chemoradiotherapy.

## Graphical abstract

Bone Marrow Sparing Intensity Modulated Radiation Therapy is a feasible option for cervical cancer patients treated with extended‐field radiation therapy, it may reduce hematologic toxicity and ensure the continuity of concurrent chemoradiotherapy.

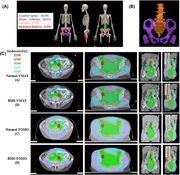

## 1 INTRODUCTION

Cervical cancer is a major public health concern worldwide.^1^ Despite the introduction of cervical cancer screening and the human papillomavirus (HPV) vaccine, cervical cancer remains a significant health issue and tends to affect younger individuals in China.^2^ To date, in cases of locally advanced cervical cancer (International Federation of Gynecology and Obstetrics [FIGO] IIB–IVA), the integration of chemotherapy with radiotherapy remains the gold‐standard treatment strategy.

**FIGURE 1:**
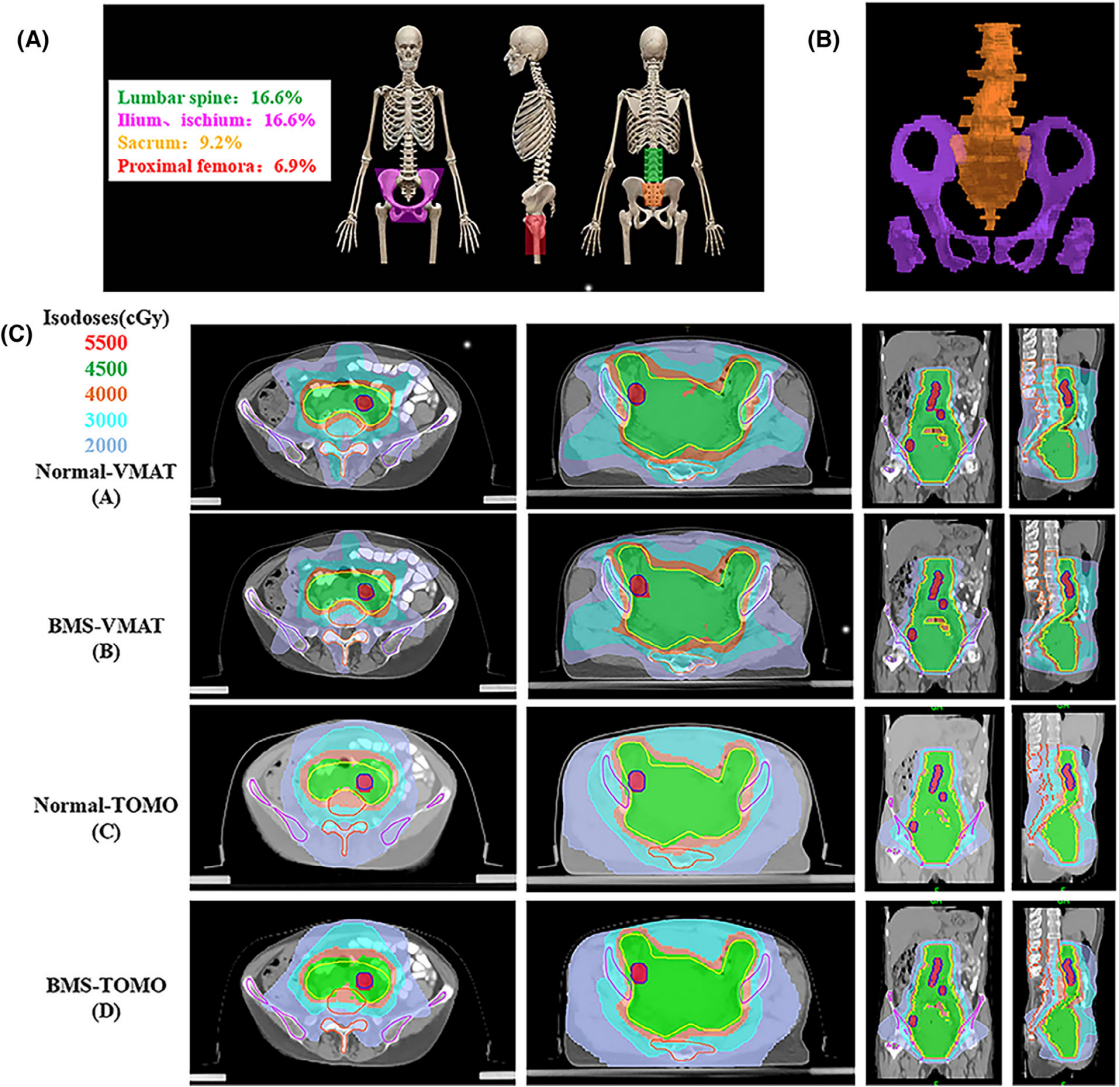
(A) Distribution of most functional bone marrow in the radiation field of conventional EFRT in cervical cancer; (B) Typical figures showing contours for bone marrow cavity in EFRT of LS (orange) and OC (purple). (C) Typical dose distributions of the four plans in cervical cancer: normal‐VMAT, BMS‐VMAT, normal‐TOMO, and BMS‐VMAT plans. EFRT, extended‐field radiation therapy; LS, lumbosacral spine; OC, os coxae; VMAT, volume‐modulated arc therapy; BMS, bone marrow sparing; TOMO, helical tomotherapy.

Pelvic lymph node (PLN) and para‐aortic lymph node (PALN) metastases are important prognostic factors,^3^, ^4^, ^5^ and the FIGO staging system for uterine cervical cancer has allowed the involvement of node status since 2018.^6^ At present, extended‐field (pelvic and para‐aortic) radiotherapy (EFRT) is indicated for patients with positive common iliac or PALNs^7^ and recommended for patients with multiple positive PLNs. Cisplatin‐based EFRT is the standard treatment for these patients. However, this approach is associated with a higher incidence of hematological and gastrointestinal toxicities compared to pelvic radiotherapy alone.^8^, ^9^ This is largely attributable to the fact that approximately 50% of active bone marrow (BM) is situated within the radiation field of conventional EFRT for cervical cancer (Figure 1A). Acute grade 3–4 hematological toxicities can result in missed cycles of concurrent chemotherapy, delays in treatment, and deterioration of clinical outcomes.^10^ Therefore, reducing hematological toxicity during concomitant cisplatin‐based EFRT is an important goal for radiation therapists.

Our previous dosimetric analysis has demonstrated that the bone marrow‐sparing (BMS) intensity‐modulated radiation therapy (IMRT) plan, which included the lumbosacral spine (LS) and os coxae (OC) as separate dose–volume constraints, significantly reduced the mean volume of BM receiving ≥10 Gy, ≥20 Gy, ≥30 Gy, and ≥40 Gy in patients with FIGO stage IIB cervical cancer. This approach may be a promising therapeutic option for reducing hematological toxicity in patients with cervical cancer.^11^ Therefore, this study aimed to evaluate the dosimetric quality and hematological toxicity of BMS‐IMRT in patients with FIGO stage IIIC cervical cancer receiving EFRT.

## 2 METHODS

### 2.1 Patient selection and characteristics

Clinical data of 84 patients with biopsy‐confirmed cervical cancer recruited between January 2019 and January 2022 were retrospectively analyzed. Patients were administered concurrent cisplatin‐based EFRT owing to the presence of positive common iliac or PALNs. Clinical staging was performed according to the updated 2018 FIGO staging classification. To assess whether PLNs or PALNs were involved, imaging examinations were performed. The imaging modalities employed included computed tomography (CT) (17.9%), magnetic resonance imaging (52.4%), and positron emission tomography (PET) (29.8%). The eligibility criteria for our study were as follows: (1) radiologically confirmed clinical FIGO stage IIIC cervical cancer; (2) pathologically confirmed cervical squamous cell carcinoma or adenocarcinoma; (3) concurrent cisplatin‐based EFRT was administered at our hospital, and the clinical data were thoroughly collected; (4) no distant metastasis; (5) no prior history of other malignancies; (6) received either a volume‐modulated arc therapy (VMAT) plan or a helical tomotherapy (TOMO) plan; and (7) had not received any radiotherapy, chemotherapy, or immunotherapy prior to radiotherapy initiation. The study was approved by the Institutional Research Medical Ethics Committee of Harbin Medical University Cancer Hospital.

### 2.2 Treatment regimen

All patients underwent extended‐field external beam radiotherapy (pelvic + para‐aortic), concurrent chemotherapy (cisplatin, 30–40 mg/m^2^ weekly), and brachytherapy. Before initiating radiation treatment, individuals were positioned supine and stabilized using custom‐molded thermoplastic immobilization systems. They were then scanned in the head‐first position using a Philips 16‐slice Brilliance big‐bore CT scanner with a slice thickness of 5 mm. Before the simulation, all patients were instructed to have a full bladder and empty rectum and to undergo bowel preparation with meglumine diatrizoate.

Targets and organs at risk (OARs) were delineated according to the guidelines stipulated in the International Commission on Radiation Units and Measurements reports Report 62 and Radiation Therapy Oncology Group 0418 protocol. The gross tumor volume (GTV) included positive lymph nodes. The clinical target volume (CTV) encompassed the pelvic and para‐aortic regions, which were delineated as zones that may contain potential microscopic diseases. These include the GTV, cervix, parametria, uterus, uterosacral ligaments, and a portion of the vagina (depending on the extent of the primary tumor), as well as the pelvic and para‐aortic lymphatic drainage areas (para‐aortic, common iliac, external iliac, internal iliac, obturator, and presacral lymph nodes). The superior boundary of the CTV was placed at L1/L2. Using image‐guided radiotherapy techniques, the planning target volume (PTV) was defined as the CTV with an additional 5 mm margin, whereas the planning gross target volume (PGTV) was delineated as the GTV covering the positive lymph nodes, also with a 5 mm margin. A radiation dose of 45 Gy was prescribed to at least 95% of the PTV and delivered in 25 daily fractions via VMAT or TOMO. A minimum of 95% of the PGTV was escalated to 61.6 Gy in 28 daily fractions using the simultaneous integrated boost and late‐course boost (LCB) techniques. For patients presenting with bulky parametrial sidewall disease, an additional parametrial boost of 6 Gy was administered using the LCB. Furthermore, patients underwent brachytherapy using high‐dose‐rate Ir‐192 sources, with a total dose of 30–36 Gy delivered to each A point in 5–6 fractions, administered either once or twice weekly.

The bladder, small intestine, rectum, femoral head, kidneys, spinal cord, BM, OC, and LS were routinely delineated as OARs. Consistent with our previous research,^11^ we delineated the marrow cavity of the intramedullary low‐density area based on the bone window in the CT simulation. This included the OC, defined as the region extending from the iliac crests to the ischial tuberosities, encompassing the ilium, pubis, ischium, and acetabula, but excluding the femoral heads, and the LS, which extends 2 cm above the upper boundary of the PTV to the coccyx. The contours of the marrow cavity are illustrated in Figure 1B, and the dose–volume constraints for the OARs are presented in Table 1.

**TABLE 1:** Dose–volume constraints of the organs at risk in cervical cancer.

Structures	Dose–volume constraints
Kidney	V_20_< 30%, D_mean_ < 12 Gy
Small bowel	V_40_ < 30%, D_max_ < 52 Gy
Rectum	V_40_ < 40%, D_max_ < 52 Gy
Bladder	V_40_ < 40 %
Femoral head	V_40_ < 5 %
Spinal cord	D_max_ < 40 Gy
OC	V_10_ < 80%, V_20_ < 50 %, V_30_ < 30%
LS	V_10_ < 90%, V_20_ < 70 %, V_30_ < 40%

Abbreviations: LS, lumbosacral spine; OC, os coxae.

### 2.3 Radiotherapy techniques

The VMAT plans were generated using Monaco 5.11 planning system. These plans utilized 6 MV X‐ray beams delivered by the Elekta Versa HD linear accelerator, with dose calculations performed using the Monte Carlo algorithm. Two 360° arc fields were designed for the VMAT, with each arc containing 200 control points. TOMO plans were generated using a tomotherapy planning system (Accuray Inc., Madison, WI, USA), employing 6 MV photon beams. The beamlet calculation parameters included a pitch value of 0.287, field width of 2.5 cm, modulation factor of 3, and normal dose calculation grid. We also redesigned 15 patients’ plan in BMS‐VMAT, BMS‐TOMO, Normal‐VAMT, and Normal‐TOMO to compare dosimetric difference. The PTV, PGTV, and OARs parameters were recorded after treatment planning completion. Target delineation for all patients, including dosimetric and hematological toxicity analyses, was performed by the same physician. In addition, the treatment plans were performed by a single physicist to ensure consistency and accuracy.

### 2.4 Evaluation of toxicity and adverse effects

All patients underwent weekly assessments for acute hematological toxicities during the course of treatment as well as at one and three months after treatment completion. Treatment‐related toxicities were classified according to the Common Terminology Criteria for Adverse Events (CTCAE v5.0). Severe toxic reactions were characterized as harmful manifestations observed throughout the clinical intervention phase or during the subsequent 3‐month monitoring window following therapeutic cessation.

### 2.5 Statistical analysis

To maintain consistency in the outcome evaluation, all therapeutic regimens were adjusted to ensure that 95% of the PTV was covered with a dose of 45 Gy. Following this standardization, dose–volume histogram metrics corresponding to the PTV, PGTV, and OARs were compared using a paired *t*‐test analysis. Hematological toxicity incidence rates across patient subgroups stratified by clinicopathological characteristics and therapeutic variables were analyzed using nonparametric statistical methods. The Mann–Whitney U test was applied to ordinal variables, and Pearson's chi‐square test was employed for categorical comparisons. Multivariate logistic regression analysis with covariate adjustment was performed to evaluate the association between hematologic adverse events (AEs) and the EFRT regimen (BM‐sparing or not).

## 3 RESULTS

### 3.1 Patient characteristics

A total of 84 participants fulfilled the inclusion criteria, with a median age of 55 years (range, 29–73 years). All participants received EFRT and had common iliac or para‐aortic lymph node metastases: 85.7% (72/84) had common iliac and 14.3% (12/84) had para‐aortic metastases. Of the 84 patients, 52 (61.9%) had tumors exceeding 4 cm in size. Patients with elevated SccAg levels >10 ng/ml prior to treatment constituted 39.3% of the study population (33/84). The clinical and pathological features of the patients are shown in Table 2.

**TABLE 2:** Clinicopathological characteristics at baseline in the BMS and control groups.

Variable	BMS group (*N* = 40)	Control group (*N* = 44)	*P*‐value
Age, (years)			0.160
≤45	2 (5%)	7 (15.9%)	
>45	38 (95%)	37 (84.1%)	
Clinical stage			0.040^a^
IIIC1r	31 (77.5%)	41 (93.2%)	
IIIC2r	9 (22.5%)	3 (6.8%)	
Pathology			0.224
Squamous	38 (95%)	44 (100%)	
Adenocarcinoma	2 (5%)	0 (0%)	
Chemotherapy cycles			0.261
1–4	10 (25%)	16 (36.4)	
5–6	30 (75%)	28 (63.6)	
SCC‐Ag value,(ng/ml)			0.053
≤10	27 (67.5%)	22 (50%)	
>10	11 (27.5%)	22 (50%)	
N/A	2 (5.0%)	0 (0%)	
Tumor size,(cm)			0.314
≤4	13 (32.5%)	19 (43.2%)	
>4	27 (67.5%)	25 (56.8%)	
Original HGB,(g/dL)			1.000
≤9	1 (2.5%)	2 (4.5%)	
>9	39 (97.5%)	42 (95.5%)	

Abbreviations: HGB, hemoglobin; BMS, bone marrow sparing.

* *P* value was computed using the chi‐square test.

^a^ Statistically significant

### 3.2 Dosimetric comparison

**FIGURE 2:**
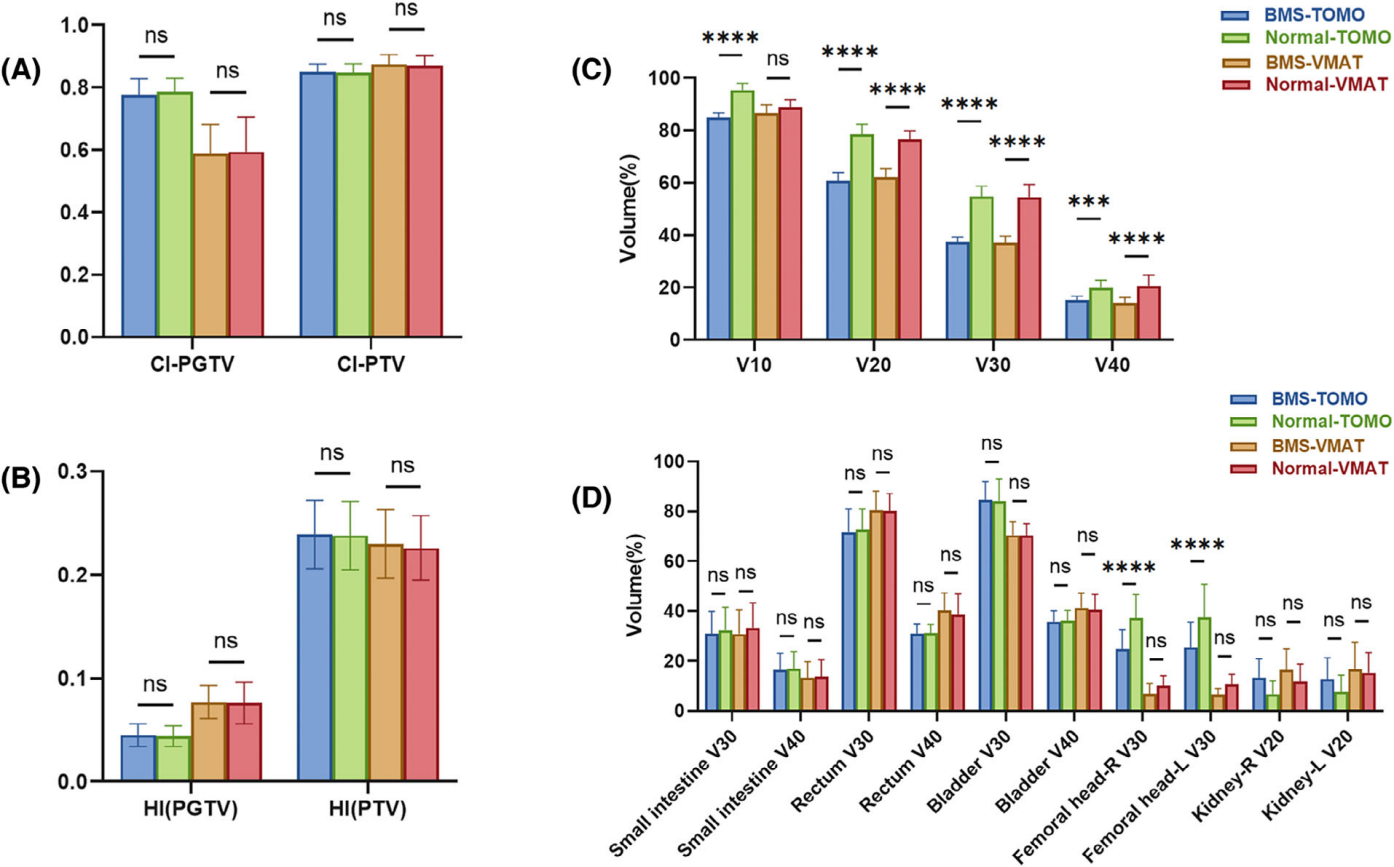
The histogram shows the difference in dosimetric parameters of PTV and OARs among the two BMS‐IMRT (VMAT and TOMO) and two normal IMRT plans (VMAT and TOMO): (A) conformity index (CI) and (B) homogeneity index (HI) for planning target volume (PTV) and planning gross target volume (PGTV); Dose–volume parameters and comparisons of (C) BM and (D) other OARs. ***P<0.001, ****P<0.0001. ns: *P* > 0.05, no statistical significance. PTV, planning target volume; OAR, organs at risk, BMS‐IMRT, bone marrow‐sparing intensity‐modulated radiation therapy; VMAT, volume‐modulated arc therapy; TOMO, helical tomotherapy.

**FIGURE 3:**
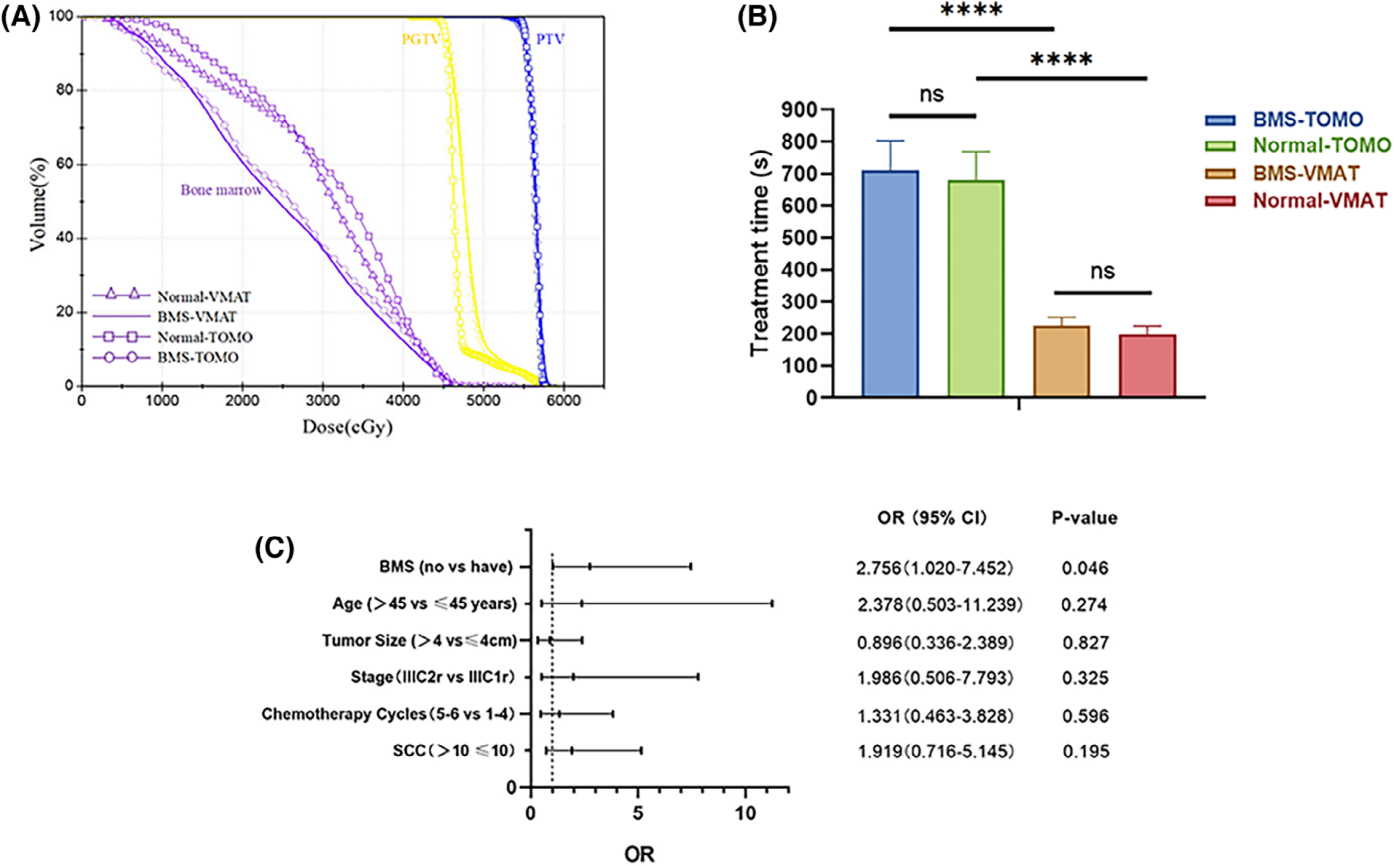
(A) Dose–volume histograms (DVHs) of PTV, PGTV, and bone marrow in different plans. (B) Comparison of treatment time among different plans. (C) Multivariate logistic regression analyses of clinicopathological variables associated with grade 3 or 4 acute hematological toxicity. ****P < 0.0001. ns: *P* > 0.05, no statistical significance.

The original treatment plans exhibited outstanding coverage of the target volume. Specifically, 95% of the PTV and PGTV was exposed to radiation doses of 45 and 55 Gy, respectively. The mean volume measurements of PTV and PGTV were 1355.82±263.02 cubic centimeters (cc) and 46.29±46.9 cc, respectively. The measured mean volume of the entire BM was 811.39 ± 127.53 cc. Specifically, the mean volumes of the LS and OC regions were 418.23 ± 127.48 cc and 419.39 ± 75.89 cc, respectively. The dosimetric parameters of BM and comparisons between the two BMS‐IMRT plans (VMAT and TOMO) and the two conventional IMRT plans (VMAT and TOMO) are presented in Table 3 and Figures 2C and 3A. Within the two BMS‐IMRT groups, a notable decline was observed in both the mean volume of BM exposed to radiation doses of 10, 20, 30, and 40 Gy, and the average radiation dose. The following are the specific decreases in volume: 10.88% and 2.57% for V_10_ (BMS‐TOMO vs. normal TOMO: 84.99±1.63% vs. 95.31±2.62%, *P*<0.001; BMS‐VMAT vs. normal VMAT: 86.60±3.16% vs. 88.88±2.74%, *P* *=* 0.005), 22.58% and 19.10% for V_20_ (BMS‐TOMO vs. normal TOMO: 60.68±3.24% vs. 78.38±3.89%, *P*<0.001; BMS‐VMAT vs. normal VMAT: 62.05±3.29% vs. 76.70±3.15%, *P*<0.001), 31.66% and 31.86% for V_30_ (BMS‐TOMO vs. normal TOMO: 37.41±1.84% vs. 54.74±3.96%, *P*<0.001; BMS‐VMAT vs. normal VMAT: 37.04±2.56% vs. 54.36±4.99%, *P*<0.001), 23.91% and 30.96% for V_40_ (BMS‐TOMO vs. normal TOMO: 15.21±1.51% vs. 19.99±2.83%, *P*<0.001; BMS‐VMAT vs. normal VMAT: 14.16±2.03% vs. 20.51±4.22%, *P*<0.001). Moreover, significant mean dose reductions were observed in the BM, reaching 15.10% and 13.00% respectively, (BMS‐TOMO vs. normal TOMO: 25.31±0.85 vs. 29.81±1.12 Gy, *P*<0.001; BMS‐VMAT vs. normal VMAT: 25.00±0.86 vs. 29.09±1.25 Gy, *P*<0.001). Compared with the normal IMRT plans, BMS‐IMRT showed better BM protection in both the VMAT and TOMO plans. Therefore, BMS‐IMRT is a promising treatment modality for reducing hematological toxicity in patients diagnosed with FIGO stage IIIC cervical cancer who are currently receiving EFRT.

**TABLE 3:** Dosimetric parameters and comparisons of PGTV, PTV, and OARs among the two BMS‐IMRT and two normal‐IMRT plans.

					*P*‐value		
	BMS‐VMAT	Normal‐VMAT	BMS‐TOMO	Normal‐TOMO	BMS‐VMAT VS Normal‐VMAT	BMS‐TOMO VS Normal‐TOMO	BMS‐VMAT VS BMS‐TOMO
PGTV							
Dmean (Gy)	56.86±0.52	56.81±0.61	56.57±0.42	56.59±0.44	0.318	0.718	0.077^a^
HI	0.077±0.016	0.076±0.020	0.045±0.011	0.044±0.010	0.704	0.560	<0.001^a^
CI	0.603±0.100	0.609±0.111	0.777±0.050	0.785±0.044	0.572	0.626	<0.001^a^
PTV							
Dmean (Gy)	48.64±0.82	48.71±0.91	47.03±0.69	47.08±0.83	0.261	0.430	<0.001^a^
HI	0.230±0.033	0.226±0.031	0.239±0.033	0.238±0.033	0.141	0.552	0.130
CI	0.830±0.033	0.824±0.045	0.851±0.024	0.847±0.029	0.432	0.465	0.014^a^
Bone marrow							
V10 (%)	86.60±3.16	88.88±2.74	84.99±1.63	95.31±2.62	0.005^a^	<0.001^a^	0.059
V20 (%)	62.05±3.29	76.70±3.15	60.68±3.24	78.38±3.89	<0.001^a^	<0.001^a^	0.101
V30 (%)	37.04±2.56	54.36±4.99	37.41±1.84	54.74±3.96	<0.001^a^	<0.001^a^	0.445
V40 (%)	14.16±2.03	20.51±4.22	15.21±1.51	19.99±2.83	<0.001^a^	<0.001^a^	0.032^a^
Dmean (Gy)	25.00±0.86	29.09±1.25	25.31±0.85	29.81±1.12	<0.001^a^	<0.001^a^	0.104
Lumbosacral spine (LS)							
V10 (%)	92.34±4.85	95.25±5.26	89.61±1.26	97.91±3.50	<0.001^a^	<0.001^a^	0.025^a^
V20 (%)	69.94±5.09	90.07±9.74	69.45±2.52	89.67±9.51	<0.001^a^	<0.001^a^	0.714
V30 (%)	42.08±3.85	67.70±11.25	43.01±1.28	67.33±9.82	<0.001^a^	<0.001^a^	0.393
V40 (%)	17.95±2.78	26.85±6.70	18.44±2.00	26.10±5.74	<0.001^a^	<0.001^a^	0.427
Dmean (Gy)	27.23±1.34	32.97±29.96	27.27±0.72	33.00±2.76	<0.001^a^	<0.001^a^	0.900
Os coxae							
V10 (%)	80.64±3.78	83.52±4.95	79.98±0.40	92.69±4.15	0.011^a^	<0.001^a^	0.517
V20 (%)	53.45±2.19	63.22±8.06	51.46±1.05	66.86±6.38	<0.001^a^	<0.001^a^	0.007^a^
V30 (%)	31.23±2.00	40.73±9.30	31.04±1.94	41.94±7.11	0.002^a^	<0.001^a^	0.637
V40 (%)	10.19±1.77	14.16±6.13	11.28±1.78	13.63±3.80	0.010^a^	0.013^a^	0.013^a^
Dmean (Gy)	22.56±0.69	25.16±2.59	23.11±0.42	26.50±1.64	0.002^a^	<0.001^a^	0.002^a^
Small intestine							
V10 (%)	84.18±10.92	83.43±10.67	91.85±9.95	91.60±9.84	0.283	0.426	<0.001^a^
V20 (%)	54.14±11.72	53.14±10.54	55.57±10.72	55.92±10.09	0.202	0.514	0.325
V30 (%)	30.89±9.62	33.17±10.20	31.00±8.95	32.37±9.13	<0.001^a^	<0.001^a^	0.871
V40 (%)	13.33±6.34	13.72±6.76	16.46±6.68	16.87±6.82	0.158	0.127	0.001^a^
Dmean (Gy)	23.19±3.87	23.20±3.76	24.31±3.64	24.46±3.58	0.979	0.052	<0.001^a^
Rectum							
V10 (%)	95.13±6.77	94.98±6.98	97.47±5.23	97.35±5.40	0.546	0.479	0.001^a^
V20 (%)	91.53±7.95	91.49±7.97	93.24±7.25	93.01±7.07	0.777	0.608	0.024^a^
V30 (%)	80.44±7.72	80.23±6.88	71.71±9.24	72.59±8.37	0.893	0.457	0.007^a^
V40 (%)	40.37±6.88	38.60±8.27	30.92±4.01	31.27±3.55	0.327	0.578	<0.001^a^
Dmean (Gy)	35.50±2.11	37.30±8.63	34.24±1.97	34.35±1.80	0.418	0.598	0.006^a^
Bladder							
V10 (%)	100.00±0.00	100.00±0.00	100.00±0.00	100.00±0.00	–	–	–
V20 (%)	94.50±6.17	94.76±6.57	100.00±0.00	99.52±1.81	0.649	0.318	0.004^a^
V30 (%)	70.47±5.38	70.19±4.91	84.73±7.34	84.21±8.87	0.741	0.673	<0.001^a^
V40 (%)	41.32±5.88	40.60±6.13	35.81±4.37	36.09±4.32	0.244	0.640	<0.001^a^
Dmean (Gy)	35.95±0.89	35.81±1.00	37.32±1.05	37.22±1.20	0.445	0.593	0.007^a^
Spinal cord							
Dmax (Gy)	23.80±5.18	35.79±3.28	35.81±5.33	32.21±2.50	<0.001^a^	<0.001^a^	0.061
Femoral head‐L							
V10 (%)	92.48±8.77	94.28±6.22	98.36±2.37	100.00±0.00	0.411	0.018^a^	0.028^a^
V20 (%)	34.98±8.85	37.50±6.13	68.23±13.22	82.07±14.31	0.125	<0.001^a^	<0.001^a^
V30 (%)	6.54±2.48	10.68±4.04	25.38±10.26	37.47±13.23	0.001^a^	<0.001^a^	<0.001^a^
V40 (%)	0.16±0.33	0.49±0.89	0.61±1.06	0.32±0.50	0.071	0.191	0.132
Dmean (Gy)	18.15±1.72	19.16±1.13	24.31±2.52	27.02±2.59	0.008^a^	<0.001^a^	<0.001^a^
Femoral head‐R							
V10 (%)	90.19±8.47	91.36±8.18	98.31±2.85	100.00±0.00	0.566	0.038^a^	0.001^a^
V20 (%)	34.46±10.06	38.01±4.95	68.47±13.45	83.75±11.04	0.218	<0.001^a^	<0.001^a^
V30 (%)	6.67±4.28	10.16±3.99	24.64±7.89	37.21±9.44	0.040^a^	<0.001^a^	<0.001^a^
V40 (%)	0.07±0.15	0.37±0.55	0.43±0.65	0.49±1.25	0.043^a^	0.771	0.035^a^
Dmean (Gy)	17.95±1.84	18.82±1.16	24.25±2.18	27.19±1.89	0.131	<0.001^a^	<0.001^a^
Kidney‐L							
V10 (%)	46.02±23.20	46.84±23.81	57.95±28.26	56.73±27.51	0.372	0.134	<0.001^a^
V20 (%)	16.72±10.83	15.21±8.20	12.70±8.60	7.42±6.98	0.361	0.001^a^	0.058
V30 (%)	2.63±3.50	1.20±1.56	0.82±1.11	0.57±0.98	0.032^a^	0.158	0.026^a^
V40 (%)	0.14±0.33	0.03±0.10	0.02±0.04	0.02±0.05	0.195	1.000	0.139
Dmean (Gy)	10.60±4.43	10.41±4.20	11.31±4.69	10.58±4.31	0.280	0.004^a^	0.042^a^
Kidney‐R							
V10 (%)	43.55±22.09	42.36±23.82	54.78±27.81	52.36±26.91	0.328	0.003^a^	<0.001^a^
V20 (%)	16.28±8.48	11.63±7.18	13.28±7.57	6.62±5.31	<0.001^a^	<0.001^a^	0.029^a^
V30 (%)	2.07±2.15	0.66±0.87	0.90±1.13	0.33±0.56	0.003^a^	0.003^a^	0.016^a^
V40 (%)	0.02±0.07	0.01±0.03	‐	‐	0.334	‐	0.334
Dmean (Gy)	10.07±4.05	9.35±3.95	10.88±4.50	9.82±4.03	0.001^a^	<0.001^a^	0.009^a^

Abbreviations: BMS, bone marrow‐sparing; PGTV, planning gross target volume; PTV, planning target volume; TOMO, helical tomotherapy; VMAT, volume‐modulated arc therapy.

*The P value was computed using a paired t test.

^a^ Statistically significant.

As depicted in Figures 2A and 2B and presented in Table 3, when evaluating the ability to attain a homogeneous and conformal dose distribution for the PTV/PGTV, the BMS‐IMRT plans displayed values of HI, CI, and D_mean_ that were comparable to those of the normal IMRT plans. This result demonstrated that BMS‐IMRT did not affect the target coverage of the PTV while protecting the BM and indicated the feasibility of its clinical application (Figure 1C).

In terms of other OARs, both the BMS‐IMRT and normal‐IMRT plans resulted in sufficient sparing of OARs. As shown in Figure 2D, BMS‐IMRT plans achieved comparable V_30_ and V_40_ for the small intestine, rectum, and bladder. Concurrently, they lead to a reduction in the V_30_ of the femoral heads, causing an elevation in the V_20_ of the kidneys. When compared with the normal IMRT plans in both TOMO and VMAT, although the BMS‐IMRT plan increased the V_20_ of the kidneys, the actual dose delivered was still within the predefined dose–volume constraints for the kidneys (V_20_ <30%, D_mean_ <12 Gy).

In addition, we also compared the difference between BMS‐TOMO and BMS‐VMAT plans, we discovered the BMS‐TOMO plans offered a significant benefit with a lower HI (BMS‐TOMO vs. BMS‐VMAT: 0.045±0.011 vs. 0.077±0.016, *P*<0.001) and higher CI (BMS‐TOMO vs. BMS‐VMAT: 0.777±0.050 vs. 0.603±0.100, *P*<0.001) were observed in the PGTV coverage, which is a small volume target. In terms of PTV coverage, BMS‐TOMO also exhibited better CI (BMS‐TOMO vs. BMS‐VMAT: 0.851±0.024 vs. 0.830±0.033, *P* = 0.014) and had similar HI, compared with BMS‐VAMT (Figures 1, 2, and 2B). Furthermore, the BMS‐TOMO plans demonstrated a substantial decrease in the volume of the bladder and rectum receiving a dose of 40 Gy (V_40_) compared with the BMS‐VMAT plans. Specifically, for the bladder, the V_40_ was 35.81% ± 4.37% for BMS‐TOMO, compared to 41.32% ± 5.88% for BMS‐VMAT (*P*<0.001). Similarly, for the rectum, V_40_ was 30.92% ± 4.01% for BMS‐TOMO, compared to 40.37% ± 6.88% for BMS‐VMAT (*P*<0.001). These results indicate superior sparing of the bladder and rectum with BMS‐TOMO (Figure 2D). In terms of BMS, both TOMO and VMAT demonstrated similar results in both high‐dose and low‐dose–volume areas, BMS‐VMAT only showed some advantage in V_40_ (BMS‐VMAT vs. BMS‐TOMO: 14.16±2.03 vs. 15.21±1.51, *P* = 0.032) (Figure 2C).

Treatment time was also evaluated, (Table 4 and Figure 3B). When compared with normal IMRT plans, the BMS‐IMRT plans had a longer mean treatment time (BMS‐TOMO vs. normal TOMO: 711.20±91.82 s vs. 680.40±89.02 s, *P*<0.001; BMS‐VMAT vs. normal VMAT: 225.95±25.52 s vs. 199.12±25.40 s, *P*<0.001). In the BMS‐IMRT plans, the mean treatment time of VMAT was significantly shorter than that of TOMO (BMS‐VMAT vs. BMS‐TOMO: 225.95±25.52 s vs. 711.20±91.82 s, *P*<0.001).

**TABLE 4:** Treatment time in different plans.

					*P*‐value		
	BMS‐VMAT	Normal‐VMAT	BMS‐TOMO	Normal‐TOMO	BMS‐VMAT VS normal‐VMAT	BMS‐TOMO VS normal‐TOMO	BMS‐VMAT VS normal‐TOMO
Treatment time (s)	225.95±25.52	199.12±25.40	711.20±91.82	680.40±89.02	0.001^a^	<0.001^a^	<0.001^a^

Abbreviations: BMS, bone marrow‐sparing; VMAT, volume‐modulated arc therapy; TOMO, helical tomotherapy.

*The P value was computed using a paired *t* test.

^a^ Statistically significant

### 3.3 Hematologic AEs

The occurrence of grades ≥2 and ≥3 acute hematological toxicity is presented in Table 5. Most acute hematological toxicities were reversible, and no treatment‐related deaths were reported.

**TABLE 5:** Incidence of grade ≥ 2 and grade ≥ 3 acute hematological toxicity in the BMS and control groups.

Toxicity	BMS group *n* (%)	Grade ≥ 2 Control group *n* (%)	*P*‐value	BMS group *n* (%)	Grade ≥ 3 Control group *n* (%)	*P*‐value
HT	34 (85)	39 (88.6)	0.622	15 (37.5)	27 (61.4)	0.029^a^
Leukopenia	34 (85)	36 (81.8)	0.698	13 (32.5)	25 (56.8)	0.026^a^
Neutropenia	18 (45)	31 (70.5)	0.033^a^	12 (30)	20 (45.5)	0.214
Thrombocytopenia	10 (25)	8 (18.2)	0.450	2 (5)	4 (9.1)	0.470
Anemia	8 (20)	21 (47.7)	0.008^a^	2 (5)	3 (6.8)	0.727

Abbreviations: BMS, bone marrow sparing; HT, hematological toxicity.

* The *P* value of grade ≥ 2 and grade ≥ 3 HT was computed by the chi‐square test. The *P* value of leukopenia, neutropenia, thrombocytopenia, and anemia was computed using the Mann–Whitney U rank test.

^a^ Statistically significant.

In the BMS‐IMRT group comprising 40 patients, 15 (37.5%) experienced grade 3 or 4 acute adverse effects. Specifically, 13 patients (32.5%) developed leukopenia, 12 (30.0%) developed neutropenia, two (5.0%) developed anemia, and two (5.0%) developed thrombocytopenia. Among the 44 patients in the normal‐IMRT group, 27 (61.4%) experienced grade 3 or grade 4 acute toxicities: 25 (56.8%) had leukopenia, 20 (45.4%) had neutropenia, 3 (6.8%) had anemia, and 4 (9.1%) had thrombocytopenia. The occurrence frequencies of ≥grade 3 acute hematological toxicity and leukopenia were remarkably greater in the normal‐IMRT group than in the BMS‐IMRT group (37.5% vs. 61.4%, *P* = 0.029; 32.5% vs. 56.8%, *P* = 0.026). The occurrence rates of ≥grade 2 neutropenia and anemia were lower in the BMS‐IMRT group than in the normal‐IMRT group (45% vs. 70.5%, *P* = 0.033; 20% vs. 47.7%, *P* = 0.008). The incidence of grades ≥2 and ≥3 thrombocytopenia did not show a significant difference. Additionally, the association between clinicopathological characteristics and severe hematologic adverse events (CTCAE v5.0 grades 3–4) was investigated using chi‐square tests and multivariate logistic regression models. As shown in Figure 3C and Table 6, BMS‐IMRT was associated with a decreased risk of grade 3 acute toxicities.

**TABLE 6:** Incidence of different grades of acute hematological toxicity in different groups.

Variable	G0–2, *n* (%)	G3–4, *n* (%)	*P*‐value
Age (years)			
≤45	5 (11.9)	4 (9.5)	1.000
>45	37 (88.1)	38 (90.5)	
Tumor size (cm)			
≤4	15 (35.7)	17 (40.5)	0.653
>4	27 (64.3)	25 (59.5)	
Pathologic types			
Squamous	40 (95.2)	42 (100)	0.494
Adenocarcinoma	2 (4.8)	0 (0)	
Stage			
IIIC1r	37 (88.1)	35 (83.3)	0.533
IIIC2r	5 (11.9)	7 (16.7)	
Chemotherapy cycles			
1–4	13 (31)	13 (31)	1.000
5–6	29 (69)	29 (69)	
SCC‐Ag value,(ng/ml)			
≤10	27 (64.3)	22 (52.4)	0.163
>10	13 (31.0)	20 (47.6)	
N/A	2 (4.7)	0 (0)	
Original HB,(g/dL)			
≤9	1 (2.4)	2 (4.8)	0.557
>9	41 (97.6)	40 (95.2)	
BMS			
No	17 (40.5)	27 (64.3)	0.029^a^
Yes	25 (59.5)	15 (35.7)	

Abbreviations: BMS, bone marrow sparing; SCC, squamous cell carcinoma.

* The *P* value was computed using the chi‐square test.

^a^ Statistically significant.

## 4 DISCUSSION

Despite effective popularization of screening and HPV vaccination, an appreciable incidence of locally advanced cervical cancer (LACC) in China remains. EFRT combined with concurrent chemotherapy has become a commonly acknowledged treatment approach for patients with LACC with para‐aortic or common iliac nodes metastases.^12^, ^13^, ^14^ Although EFRT yields encouraging survival outcomes for these patients, the radiation‐related hematological toxicity resulting from the large irradiation field of EFRT and substantial irradiated volume of the BM needs to be considered.^15^, ^16^ High‐grade acute hematological toxicity poses a significant concern, as it affects approximately 60% of patients undergoing EFRT,^17^ which can lead to treatment breaks. Therefore, reducing hematological toxicity from EFRT with concurrent chemotherapy in patients with FIGO IIIC cervical cancer is of significant clinical interest and may further improve treatment efficacy.

BM is highly sensitive to irradiation. Radiation exposure significantly affects the continuous replenishment of circulating peripheral blood cells by hematopoietic stem cells residing in BM. In particular, the administration of radiation doses of 2–4 Gy over 1–3 days can substantially decrease both cellular density and proliferative activity of the BM. Another crucial factor is the volume of the BM exposed to irradiation.^18^, ^19^ The volume of BM exposed to both a low‐dose range of 10–20 Gy^20^, ^21^, ^22^, ^23^ and a high dose of 40 Gy are factors linked to ≥grade 2 hematological toxicity.^24^ Approximately half of the active BM is situated in the lumbar sacrum, pubis, ilium, ischium, and proximal femur^25^ and encompasses the radiation field of conventional EFRT in cervical cancer. Therefore, reducing BM damage during EFRT may reduce hematological toxicity in patients and improve their tolerance to treatment.

BM delineation methods in BMS‐IMRT include pelvic whole bone delineation,^26^, ^27^ BM cavity delineation,^17^, ^28^, ^29^ and active BM delineation according to the functionally active areas using PET‐CT. Previous studies centered on PET‐guided functional BM have indicated that the majority of functional BM are situated within the marrow cavity.^30^, ^31^, ^32^ Moreover, results from earlier studies have also suggested that when compared with the entire BM, IMRT plans that utilized the lumbosacral spine and hip bones as dose–volume constraints demonstrated the most effective sparing of the BM.^33^ In our previous study on BMS‐IMRT in FIGO IIB cervical cancer,^11^ we delineated low‐density intramedullary areas as BM regions and divided the pelvic BM into two distinct segments: the LS and OC. The IMRT plan for BMS, which employed LS and OC as distinct dose–volume constraints, attained the most efficient BMS. This plan resulted in the lowest BM volume for doses of ≥10 Gy, ≥20 Gy, ≥30 Gy, and ≥40 Gy. Therefore, we continued to use our previous BM delineation methods and dose–volume constraints in the current study and conducted a dosimetric study of BM‐sparing IMRT in EFRT. Compared to the normal IMRT plans, the BMS‐IMRT significantly decreased the volumes of BM exposed to doses of ≥10 Gy, ≥20 Gy, ≥30 Gy, and ≥40 Gy, without compromising the coverage of PTV and other OARs. Thus, applying BMS‐IMRT to EFRT may reduce hematological toxicity. In addition to volume dose limitations and delineation methods for BM, most studies have focused on radiotherapy techniques. Compared to conventional three‐dimensional conformal radiation therapy (3D‐CRT), IMRT achieves superior BM dose reduction through its characteristic beam configuration, employing multiple gantry angles or arcs, and demonstrating distinct dosimetric advantages for pelvic BM preservation.^34^, ^35^, ^36^ Intensity‐modulated proton therapy has also demonstrated significance and robustness in BM sparing.^37^ VMAT and TOMO are the most commonly used radiotherapy techniques for patients with cervical cancer in China. Therefore, these two radiotherapy techniques were included in our study. Both techniques were effective in sparing the BM, achieving comparable outcomes in terms of volume in both high‐ and low‐dose areas. In addition, TOMO planning achieved superior dose conformity to both the PTV and PGTV while simultaneously decreasing high‐dose irradiation volumes in the bladder and rectal tissues adjacent to the target region compared to VMAT plans; this finding is consistent with those of previous studies.^38^, ^39^ Although TOMO demonstrated better HI and CI of the target and better sparing of OARs, VMAT met our clinical demands. VMAT required a much shorter treatment time than TOMO when delivering EFRT. Therefore, VMAT may be more suitable for BMS‐IMRT in China, which is a developing country, considering its insufficient medical resources.

Recently, BMS‐IMRT has been increasingly adopted to manage diverse cancer types, and has demonstrated favorable clinical outcomes. One prospective trial on stage IB2–IIIB cervical cancer confirmed that BMS‐IMRT significantly reduced hematological toxicity. However, to date, studies on BMS‐IMRT and its hematological toxicity have been few. Therefore, this study aimed to identify the preliminary clinical advantages of BMS‐IMRT in patients with FIGO IIIC cervical cancer treated with EFRT. As previously noted, hematological toxicity is a significant concern in patients undergoing EFRT. Previous studies of normal EFRT with concurrent chemotherapy have demonstrated that grade ≥3 hematological toxicity rates can be high at 37.5%–77.5%.^9^ Our retrospective analysis showed that patients treated with BMS‐IMRT were less likely to experience hematological toxicity (leukocytes, neutrophils, hemoglobin, and platelets) than those treated with normal IMRT. Compared with the normal‐IMRT group, the BMS‐IMRT group had a lower incidence rate of grade 3 or 4 acute hematological toxicity (37.5% vs. 61.4%, *P* = 0.029). Multivariate logistic regression analyses also demonstrated that BMS‐IMRT was associated with a reduced risk of grade 3–4 acute toxicities (odds ratio, 2.756; 95% confidence interval [*CI*], 1.020–7.452; *P* = 0.046). Therefore, using BMS‐IMRT is important in treating FIGO IIIC cervical cancer, particularly in patients receiving concurrent chemotherapy.

Our study had some limitations. Since we mainly focused on acute hematological toxicity using information from the past 2 years, long‐term side effects and patient survival data after treatment were lacking. In addition, this study was a retrospective, single‐institution investigation conducted on a restricted patient cohort, resulting in imperfect matching between the two groups. Therefore, prospective clinical trials incorporating more extensive clinical data and direct dosimetric parameters of the BM are necessary to reach a consensus on the efficacy of BMS‐IMRT in reducing hematological toxicity in patients undergoing EFRT.

## 5 Conclusion

BMS‐IMRT should be administered in patients with FIGO stage IIIC cervical cancer treated with EFRT, brachytherapy, and concurrent chemotherapy. BMS‐IMRT was associated with a clear improvement in grade ≥3 hematological toxicity rates. The findings of this study will guide the optimized clinical implementation of BMS‐IMRT in patients with FIGO stage IIIC cervical cancer undergoing EFRT.

## AUTHOR CONTRIBUTIONS

SS. Y, DY. Y, and JN. W contributed to study design and interpretation. JN. W and X.Y contributed to manuscript writing, and SS. Y and LN. G contoured the target and OARs and DY. Y and XY. Hu designed the treatment plan; X.Y and WK.Y contributed to the dosimetric data collection. DM.L, QY.S, JQ.X, and X.L contributed to clinicopathologic data collection. SS. Y and DY. Y contributed to the critical manuscript revision. All authors agree with the final version and are accountable for the integrity of the published data.

## CONFLICT OF INTEREST STATEMENT

All authors declare no potential conflicts of interest.

## ETHICS APPROVAL

This study complied with the Declaration of Helsinki and was approved by the Ethics Committee of Harbin Medical University Cancer Hospital (Harbin, China). All patients provided written informed consent for publication of their images/data.

## Data Availability

The data supporting the findings of this study are available on request from the corresponding author.
